# Tissue-Derived Stem and Progenitor Cells

**DOI:** 10.4061/2010/824876

**Published:** 2009-10-28

**Authors:** Leora J. Tesche, David A. Gerber

**Affiliations:** Department of Surgery, University of North Carolina School of Medicine, Chapel Hill, NC 27599-7211, USA

## Abstract

The characterization and isolation of various stem cell populations, from embryonic through tissue-derived stem cells, have led a rapid growth in the field of stem cell research. These research efforts have often been interrelated as to the markers that identify a select cell population are frequently analyzed to determine their expression in cells of distinct organs/tissues. In this review, we will expand the current state of research involving select tissue-derived stem cell populations including the liver, central nervous system, and cardiac tissues as examples of the success and challenges in this field of research. Lastly, the challenges of clinical therapies will be discussed as it applies to these unique cell populations.

## 1. Introduction

Stem cells are broadly defined as cells capable of going through numerous cycles of cell division, maintaining an undifferentiated state and having the capacity to differentiate into specialized cell types. They will often go through asymmetric division where the stem cell creates a copy of itself and a daughter cell that is capable of differentiation [1]. Stem cells are further classified into three categories: totipotent, pluripotent, or multipotent somatic. A totipotent cell has the ability to form an entire organism (e.g., a fertilized egg). Pluripotent stem cells lack the ability to form extraembryonic tissue and are therefore unable to generate a fetus. Examples of pluripotent cells include embryonic stem cells which can give rise to any cell type from the three germ cell layers (i.e., endoderm, mesoderm, or ectoderm). Adult stem cells or mesenchymal stem cells (MSCs) are examples of pluripotent stem cells that are isolated from mature tissues and differentiate into other tissue types. Multipotent somatic stem cells are capable of differentiating into a variety of closely related cells within a tissue, but lack the ability to differentiate into other tissues. A much studied area of multipotent somatic stem cells is the hematopoietic stem cell (HSC) which generates daughter cells that in turn differentiate into all subpopulations of hematopoietic cells (e.g., red blood cells, platelets, etc.) [2].

Several challenges persist when investigating individual stem cell populations [3]. These hurdles include identifying unique markers for the cells, ability to isolate the cells and potential legal and ethical concerns around this field of research. There are well-documented techniques on the methods to obtain embryonic stem cells from the blastocyst and grow them in culture [4]. Subsequent work has demonstrated that these cells can be coaxed into a variety of different cellular subtypes based upon their local environment [5–8]. Challenges in embryonic stem cell research have come from both biologic and regulatory areas. The proliferative and pluripotent nature of embryonic stem cells have been associated with the development of teratomas, an obvious problem when designing clinical therapies [9]. From the regulatory perspective, there were federal laws that previously limited the use of certain embryonic stem cells; recent changes in the law have relaxed these restrictions.

Identifying and isolating somatic stem cells from mature organs is a greater challenge. Pluripotent cells are thought to exist in most adult tissues, but their low frequency and lack of identified unique cell surface markers make it difficult to isolate these cells. Maintaining these stem cells in an undifferentiated state or even directing their differentiation requires understanding the signaling pathways that naturally occur in the cells' extracellular environment (e.g., the “stem cell niche”). The local milieu provides critical signals for most stem cell populations to continue self-renewal [10]. Upon exiting the niche these cells begin to differentiate into a committed cell [11–13]. Recent work involving stem cell signaling pathways has enabled researchers to reprogram somatic cells to exhibit essential characteristics of embryonic stem cells [14–16]. This technique of harvesting accessible somatic cells (e.g., hematopoietic cells or adipocytes) and then inducing them into other tissue types is an active area of investigation (i.e., induced pluripotent stem cells). Stem cell differentiation into the functional phenotype of heart, liver, pancreas, nervous system, and gut are under active investigation. The following outline is a summary of research in stem cell biology and its potential clinical applications within select fields.

## 2. Pancreas

### 2.1. Anatomy and Function of Tissue

The human pancreas is a glandular organ that serves both endocrine and exocrine functions. The exocrine pancreas is comprised of acinar, centroacinar, and duct cells. These cells manufacture and secrete enzymes and alkaline fluids into the intestinal tract to facilitate digestion. The endocrine pancreas consists of the Islets of Langerhans which are mixed cell clusters that produce hormones for excretion into the blood stream. These cell-type-specific hormones include insulin and glucagon which are essential in glucose regulation. Other hormones produced in the Islets of Langerhans include somatostatin which is important in digestive regulation.

### 2.2. Clinical Need for Tissue Engineering

The pancreas has been implicated in many disease states [17] and plays a particularly important role in glucose regulation. Type 1 diabetes occurs secondary to multiple factors that lead to a paucity of pancreatic *β* cells within the Islets of Langerhans. This renders an individual unable to regulate blood glucose levels without the use of exogenous insulin and currently affects nearly 5 million individuals worldwide [18]. Pancreatic islet cell transplantation has been heralded as a curative treatment for these patients [19, 20]. Unfortunately the limited supply of pancreatic tissue for transplantation and ongoing immunologic issues are two major hurdles that have prevented broader success with this therapy [20].

### 2.3. Potential of Embryonic Stem Cells for Tissue

Embryologic development of the pancreas has been closely studied and known mediators and transcription factors have been identified in pancreatic development. Activin- and (fibroblast growth factor-2) FGF-2 mediated repression of sonic hedgehog expression have been implicated in pre-pancreatic development from dorsal endoderm [17]. These factors induce (pancreatic and duodenal homeobox 1) Pdx1 expression which is thought to be the master regulator for further pancreatic development [17]. The subsequent determination of insulin producing *β* cells occurs by sequential expression of the transcription factors Nkx2.2, Pax2, NKx6.1, MafQ, Pax6, and Pdx1 [21]. Knowledge of this pathway has allowed researchers to focus on developing functional insulin-producing cells from pluripotent stem cells by manipulating cell culture medium [6, 22].

Human embryonic stem cells have been successfully cultured to create pancreatic islet cells that produce insulin and C-peptide in response to glucose stimulation. Jiang et al. describe a 36-day protocol that involves mixing human embryonic stem cells (HESCs) in vitro with Pdx1, Ptf1a/p48, Activin, and FGF in a sequential fashion to drive differentiation into functional pancreatic cells [23]. In vitro C-peptide levels were measured but in vivo functionality was not studied. This protocol has been repeated among other groups with slight variations in the cell culture medium and transcription factors [24–27]. These efforts and other cell culture manipulations have fueled the debate that possible contaminants are leading to the impression of cell differentiation.

### 2.4. Potential of Adult Stem Cells for Tissue

Identification and isolation of a defined pancreatic stem cell capable of giving rise to *β* cells has been somewhat controversial. There has been an ongoing debate surrounding the presence of a persistent stem cell subpopulation versus regeneration through self-duplication of adult pancreatic betacells [21, 28–33]. Evidence suggests that a subpopulation of ductal cells is capable of differentiation but the cell numbers are small and obtaining these cells for functional investigation has been difficult [18, 34]. Current initiatives are aimed at identifying molecular markers that characterize pancreatic stem cells and facilitate their isolation.

### 2.5. Clinical Studies

Some of the most advanced clinical studies have come from the use of bone marrow-derived stem cells. Karnieli et al. demonstrated that bone marrow stem cells induced with Pdx1 and other factors can functionally resemble pancreatic cells [25]. Bone marrow-derived cells that have been induced with Pdx1 have been inserted under the renal capsule in diabetic animals and treated animals became normoglycemic after transplantation of manipulated cells [35]. Similar results have been demonstrated in mice after viral vector injection of manufactured insulin producing cells from bone-marrow stem cells [25].

Overall, the clinical need for appropriate therapies for the treatment of type 1 diabetes is clear. The number of patients that undergo pancreas or islet cell transplant remains limited relative to the number of patients afflicted with this disease. The breadth of efforts in stem cell research from embryonic stem cells through adult pluripotent cells has been somewhat successful in animal models. Translational efforts in clinical trials will be the obvious critical step in the domain of diabetes therapy.

## 3. Cardiac

### 3.1. Anatomy and Function of Tissue

The human heart is a muscular organ that is responsible for pumping oxygenated blood from the lungs to peripheral tissues. The cardiac tissue is comprised of three layers. The innermost layer is the endocardium which lines the inner chambers of the heart. Surrounding this is the myocardium which consists of cardiac myocytes (i.e., involuntary striated muscle cells). These cells contract in response to electrical stimuli. The fibrous epicardium functions as a scaffold to provide shape and form to the heart. Blood supply arrives to the cardiac tissue through the coronary vessels. 

### 3.2. Clinical Need

Heart disease is the number one cause of mortality in the United States. A myocardial infarction occurs secondary to a blockage in the coronary vessels supplying the cardiac myocytes. Myocytes die in response to the ischemic event thereby forming a scar in the tissue. Surrounding the infracted area is a region of stunned or “hibernating” myocardium. The likelihood of acutely dying from a myocardial infarct has decreased 30% over the past 20 years, because of improvements in management of heart disease. However, the number of people living with compromised heart function has nearly doubled over the same period [36]. For patients with a new diagnosis of congestive heart failure there is a mortality rate of 20% within the first year and 50% within two years [37]. The prevalence and pathophysiology of heart disease makes it a principal target for novel therapies. Areas of investigation include the role of stem cells in cardioprotection and/or remodeling after myocardial infarction. 

### 3.3. Potential of Embryonic Stem Cells for Tissue

Cardiac-myocyte-like cells have been induced from embryonic stem cells by coculture with endodermal feeder layers and/or growth factors. These induced cell populations form contractile tissue that expresses troponin and other markers demonstrated in mature cardiac myocytes [38]. Embryonic derived cardiac myocytes are currently being explored for their therapeutic potential in injured myocardium [39]. 

### 3.4. Potential of Adult Stem Cells for Tissue

Studies of heart transplant recipients demonstrate that host cells from hematopoietic origins can repopulate the new myocardium [40]. This has been used as evidence to support the idea that bone marrow derived progenitor cells (BMCs) may provide assistance to the injured heart. Cardiomyocyte progenitor cells have also been identified within the epicardium of the heart. These cells are thought to repopulate the heart in times of injury and animal models have evaluated their therapeutic efficacy after myocardial infarction [41, 42]. Bearzi et al. identified a class of human *c-kit*-positive cardiac cells that possess the fundamental properties of stem cells: they are self-renewing, clonogenic, and multipotent. When these cells were locally injected in the infarcted myocardium of immunodeficient mice the human cardiac stem cells generate a chimeric heart [41]. 

### 3.5. Clinical Studies

Large-scale studies to assess the effectiveness of bone marrow stem cell implantation after myocardial infarction include the Reinfusion of Enriched Progenitor Cells and Infarct Remodeling in Acute Myocardial Infarction (REPAIR-AMI) and Bone MarrOw Transfer to Enhance ST-elevation infarct regeneration (BOOST) trials. In the REPAIR-AMI trial, patients who experienced a myocardial infarct were randomized to receive either intracoronary infusion of bone marrow cells or placebo after traditional revascularization procedures (e.g., stenting). The primary measured outcomes were: left ventricular ejection fraction (LVEF), recurrent MI, additional revascularization procedures, heart failure, and death. LVEF improved over time in both the BMC recipient group as well as the placebo group but the improvement was significantly greater at four months in the bone marrow transplant group (*P* = .01) [43]. Death, recurrence of MI, and hospitalization when examined together were also lower in the BMC group after 1 year (*P* = .006) [43]. Given the safety profile of this treatment and the beneficial effects in patients with the most severely impaired left ventricular function, large-scale studies are warranted to examine the potential effects of this novel approach on the risk of death and complications in patients with large acute myocardial infarctions and depressed left ventricular contractile function [43].

The BOOST trial, like the REPAIR-AMI trial, included patients with an acute ST elevation myocardial infarction who underwent percutaneous coronary intervention (PCI). Patients were prospectively randomized to receive optimal medical therapy or medical therapy plus bone marrow cell transplant [44]. After 6 months, the authors observed a 0.7% absolute improvement in ejection fraction of the control group versus 6.7% improvement in the bone marrow transplant group (*P* = .0026). However, the improvement in LVEF was not sustained at 18 months [44].

Ongoing trials have investigated the delivery and timing of stem cell infusions in relation to the cardiac event. While the optimal time for transplantation has not been delineated in a randomized trial current data suggests that the most effective time of delivery may be between 5–8 days after the ischemic event [45].

Studies involving embryonic-derived cardiac myocytes have shown some functional success, but thus far have been limited to animal models [39]. Min et al. describe direct injection of embryonic derived cardiac myocytes into areas of injured heart after surgically induced myocardial infarction. At 32-week follow-up the mice who received stem cell injections demonstrated improved left ventricular function and overall survival when compared to the sham group. By histology, the stem cell group had a higher density of blood vessels surrounding the area of infarct suggesting that stem cells may enhance neovascularization in injured myocardium. 

## 4. Liver

### 4.1. Anatomy and Function of Tissue

The human liver is the body's chief metabolic organ. It is comprised of several cell types including hepatocytes, cholangiocytes, kupffer cells, stellate cells and endothelial cells. Among its many functions are the production of proteins, the regulation of blood glucose levels, and enzymatic degradation of toxins within the body.

### 4.2. Clinical Need for Tissue Engineering

Due to the liver's essential regulatory and synthetic functions, hepatic failure is a life threatening condition. Although hepatic regeneration can occur via terminally differentiated hepatocytes, this regenerative capacity remains insufficient in the majority of disease states and a patient may subsequently require liver transplantation. Due to the shortage of available organs and the need for lifelong immunosuppression, ongoing research is focused on strategies to repopulate the liver with functioning hepatocytes through cell transplantation.

### 4.3. Potential of Embryonic Stem Cells for Tissue

Embryonic stem cells serve as a potential source for cell transplantation to reconstitute the diseased liver [46]. Studies utilizing culture manipulation of embryonic stem cells have elicited transformation into “hepatocyte-like cells” [46, 47]. A challenge in working with pluripotent embryonic stem cells is the potential for some cells to remain in the undifferentiated state. When these undifferentiated cells are transplanted there is the potential for unchecked growth and teratoma formation. Current efforts aim to maximize functional capacity and stability of induced hepatocytes for transplantation [47, 48].

### 4.4. Potential of Adult Stem Cells for Tissue

Efforts to identify a somatic-derived pluripotent cell within the liver have led to the discovery of several multipotent cell types. Examples of multipotent cells include the hepatoblast and oval cell. In the fetal liver, hepatoblasts serve as precursors for hepatocytes as well as cholangiocytes. These cells are characterized by specific cell surface markers including alpha-fetoprotein (AFP), EpCAM, and cytokeratins 17 and 19 [49]. During embryogenesis, most of these cells terminally differentiate into hepatocytes or biliary cells. However, a distinct cell population maintains its capacity for self-renewal and differentiation suggesting that hepatic stem cells persist in fetal tissue [48].

In the adult liver, a bipotent progenitor cell capable of forming both liver and biliary epithelium has been identified. These cells, termed oval cells, reside in the terminal bile ducts of the adult liver, and maintain a high nuclear/cytoplasmic ratio [50–52]. They express markers of immature liver and biliary epithelium including alpha-fetoprotein (AFP) and CK19, respectively. Oval cells are found in increased number after acute injury to the liver and are thought to play a role in its regenerative capacity [51, 53, 54].

The adult derived hepatic progenitor cell is another cell type found in the liver and localized to the “Canals of Herring.” They appear distinct from the hepatoblast or the oval cell as they are encountered without a preceding injury to the tissue. Further investigations will need to be performed to understand the overlap amongst the various hepatic populations [55, 56].

### 4.5. Clinical Studies

Hepatocytes obtained from adult livers have been used in clinical trials to treat liver disease ranging from fulminant liver failure to inherited metabolic disorders [57, 58]. Unfortunately the supply and viability of these cells is limited [47]. Recent experimental studies involving adult-derived hepatic progenitor cells or embryonic stem cells may broaden the potential sources for cellular transplantation [59–62].

Initial studies using embryonic stem cell-derived hepatocytes as well as induced pluripotent cells are underway in animal models. Kumashiro et al. demonstrated improved liver function in mice with chemically induced liver injury after transplantation with embryonic stem cell-derived hepatocytes [47]. Similarly, induced pluripotent cells including those of hematopoietic, adipogenic, and bone marrow origins have been shown to improve hepatic fibrosis in select animal models [63]. While some animal studies have been initially successful, large-scale human trials involving stem cells and liver failure have yet to be realized. Current limits for these studies involve assuring stem cell stability and a safe method for transplantation.

## 5. Nervous System

### 5.1. Anatomy and Function of Tissue

The nervous system, a network of neurons and supporting cells that interpret and respond to stimuli, is divided into two compartments. The central nervous system (CNS) is comprised of the brain and spinal cord. Oligodendrocytes are cells that create the myelin sheath that surround the neurons of the CNS. Myelination protects the neurons and aids in the speed of signal transmission. The peripheral nervous system (PNS) supplies sensory and motor information to and from the extremities. Schwann cells myelinate the nerves of the peripheral nervous system. 

### 5.2. Clinical Need for Tissue Engineering

While peripheral nerves have some regenerative capacity, damage to the central nervous system usually results in permanent disability. Therefore, degenerative conditions such as Parkinson's disease and multiple sclerosis are devastating diagnoses. Similarly, traumatic spinal cord injury usually results in irreversible paralysis. Treatment for these conditions has traditionally been limited to supportive therapy. Recent investigations have demonstrated a population of pluripotent cells within the CNS that is responsible for repair and regeneration. Recent efforts have been directed towards harnessing the potential of these cells for therapeutic use.

### 5.3. Potential of Embryonic Stem Cells for Tissue

Embryonic stem cells can be induced to form neurons and other functional neural tissue. Ben-Hur et al. demonstrated that functional neuronal cells could be derived from primate embryonic stem cells using a stromal feeder coculture system for neural induction with sequential exposure to inductive signals, such as sonic hedgehog (SHH) and FGF-8. This approach controls dopaminergic specification during embryogenesis. After transplantation into immunosuppressed rats these cells maintain functional stability for 12 weeks [64]. In a primate model of Parkinson's disease Takagi et al. demonstrates stability of embryonic derived dopamanergic cells [65]. Other investigators have created and transplanted embryonic derived Oligodendrocyte Progenitor Cells (OPCs). Mice with spinal cord injuries demonstrated increased remyelination and improved locomotion after injection of embryonic derived OPCs into the injury site [66]. With these previous experimental studies, multiple sclerosis and other demyelinating diseases are being evaluated as therapeutic targets for OPCs.

### 5.4. Potential of Adult Stem Cells

During development, the brain arises from a layer of neuroepithelial stem cells that surrounds the lumen of the early neural tube. Recent investigation has demonstrated that multipotent neural stem cells continue to line the cerebral ventricles of the forebrain in the adult brain. These cells have been isolated and grown in culture. When transplanted into neural tissue, the multipotent neural stem cells differentiate into neurons and supportive neural tissue including oligodendrocytes and glial cells [67].

### 5.5. Clinical Studies

Clinical applications of embryonic and somatic derived neural stem cells are under investigation for treatment of diseases that were once thought to be irreversible (e.g., Parkinson's disease, multiple sclerosis and traumatic spinal cord injury). The following is a contemporary description of progress in this field.

Parkinson's disease results from destruction of nigrostriatal dopamine containing neurons and physiologically manifests as rigidity, bradykinesia, tremor, and postural instability. Medical therapies to increase dopaminergic function have limited efficacy due to side effects and durability of the treatment. Promising results using stem cell based therapies have been reported by a number of groups using preclinical models, but clinical safety and the long term outcomes have not been demonstrated [68]. In one study, Takagi et al. demonstrated an effective method to create dopaminergic cells from embryonic stem cells. They subsequently demonstrated stability of grafted cells as well as functional improvement in motor behavior in a primate model of Parkinson's disease [65].

Current therapeutic efforts for spinal cord injuries are directed at limiting progression of disease rather than repairing the existing damage. Isolation of neural progenitor cells from adult or embryonic tissue may represent a novel therapeutic approach [67]. In 2005, embryonic-derived Oligodendrocyte Progenitor Cells (OPCs) were transplanted into mice with spinal cord injuries. The mice that received treatment demonstrated increased remyelination and improved locomotion [66]. Multiple sclerosis and other demyelinating diseases are also being studied as therapeutic targets for OPCs.

A limitation of stem cell transplantation of neural tissue involves the low level of integration and stability of the derived cells. Therefore, transplantation of a viable number of these cells leading to durable functioning grafts will need to be addressed for the initiation of clinical trials.

## 6. Cell Survival

In all of the described research areas, one of the crucial elements involves understanding the processes that enable stem cells to regenerate or differentiate. In adult tissue there are relatively few stem cells within a given organ. Harnessing the ability to expand these cells and maintain their undifferentiated state is important before large scale therapeutics can be realized. Purifying the cells and preventing teratoma formation is paramount to ensure the safety of stem cell therapies. Initial work with the forkhead 0 (fox0) family of transcription factors suggests that these factors may be involved in cell cycle arrest, differentiation, and apoptosis [69]. Further investigations to understand these mechanisms are essential before the full potential of stem cells can be realized.

## 7. Conclusion

The field of stem cell research continues to expand with characterization and isolation of various stem cell populations from the embryonic stem cell to tissue-derived cell populations. The clinical applications of embryonic stem cells are limited by ethical concerns and the potential of teratoma formation. Pluripotent cells which persist in mature organs are also targeted for cellular transplantation or organ regeneration. Recent gains in the understanding of the stem cell niche and the signaling pathways which drive stem cell differentiation have enabled investigators to induce readily available cells such as adipocyte and hematopoietic derived cells into other tissue types.

In this review we discussed four organ systems, the pancreas, liver, heart, and neural systems. These were selected due to the magnitude of their disease burden on society. However, it should be recognized that stem cells are also under clinical investigation in the fields of plastic and reconstructive surgery [70], ophthalmology [71], and hematopoietic diseases.
